# Autonomic Nervous System Response during Light Physical Activity in Adolescents with Anorexia Nervosa Measured by Wearable Devices

**DOI:** 10.3390/s19122820

**Published:** 2019-06-24

**Authors:** Lucia Billeci, Alessandro Tonacci, Elena Brunori, Rossella Raso, Sara Calderoni, Sandra Maestro, Maria Aurora Morales

**Affiliations:** 1 Institute of Clinical Physiology, National Research Council of Italy (CNR), via Moruzzi 1, 56124 Pisa, Italy; lucia.billeci@ifc.cnr.it (L.B.); morales@ifc.cnr.it (M.A.M.); 2 IRCCS Stella Maris Foundation, Viale del Tirreno 331, 56018 Calambrone, PI, Italy; ebrunori@fsm.unipi.it (E.B.); sara.calderoni@fsm.unipi.it (S.C.); sandra.maestro@fsm.unipi.it (S.M.); 3 The BioRobotics Institute, Scuola Superiore Sant’ Anna, Viale Rinaldo Piaggio 34, 56025 Pontedera, PI, Italy; rossella.raso@santannapisa.it; 4 Department of Clinical and Experimental Medicine, University of Pisa, Via Roma 55, 56100 Pisa, Italy

**Keywords:** anorexia nervosa, autonomic function, eating disorders, exercise, heart rate, heart rate variability, wearable, wireless technologies

## Abstract

Anorexia nervosa (AN) is associated with a wide range of disturbances of the autonomic nervous system. The aim of the present study was to monitor the heart rate (HR) and the heart rate variability (HRV) during light physical activity in a group of adolescent girls with AN and in age-matched controls using a wearable, minimally obtrusive device. For the study, we enrolled a sample of 23 adolescents with AN and 17 controls. After performing a 12-lead electrocardiogram and echocardiography, we used a wearable device to record a one-lead electrocardiogram for 5 min at baseline for 5 min during light physical exercise (Task) and for 5 min during recovery. From the recording, we extracted HR and HRV indices. Among subjects with AN, the HR increased at task and decreased at recovery, whereas among controls it did not change between the test phases. HRV features showed a different trend between the two groups, with an increased low-to-high frequency ratio (LF/HF) in the AN group due to increased LF and decreased HF, differently from controls that, otherwise, slightly increased their standard deviation of NN intervals (SDNN) and the root mean square of successive differences (RMSSD). The response in the AN group during the task as compared to that of healthy adolescents suggests a possible sympathetic activation or parasympathetic withdrawal, differently from controls. This result could be related to the low energy availability associated to the excessive loss of fat and lean mass in subjects with AN, that could drive to autonomic imbalance even during light physical activity.

## 1. Introduction

Anorexia nervosa (AN) is a psychiatric disorder characterized by a disturbed body image, constant concerns over weight and shape, and an intensive fear of gaining weight, all leading to severe dietary restriction and weight loss [1]. Subjects with AN show an enhanced mortality rate as compared with age-matched healthy controls [2], which is also due to several cardiac complications, such as pericardial effusion up to cardiac tamponade, extreme bradycardia, and ventricular arrhythmias (see the recent systematic review by Giovinazzo et al. [3]).

Furthermore, AN subjects display a wide range of autonomic disturbances, mostly as a result of the massive imbalance of the parasympathetic-to-sympathetic system activity, with a general predominance of the parasympathetic tone, especially in adolescents [4,5,6,7,8,9,10,11,12,13,14,15,16]. Such abnormalities represent good predictors of cardiac mortality risk in AN [6,17,18]; therefore, their monitoring might be clinically relevant in this subset of patients.

In the assessment of the autonomic nervous system (ANS) activity, heart rate (HR), and its variability (heart rate variability, HRV) have a clear role. Indeed, it is possible to analyze the HRV for several purposes, including the evaluation of the autonomic HR control during exercise in different physiological conditions [19,20], as the ANS plays a crucial role in the cardiovascular response to acute (dynamic) exercise in humans. Respiration appears to influence changes in electrocardiographic (ECG) high frequencies (HF) during exercise, while low frequency (LF) modulation depends on blood pressure modifications [19]. In addition, it is possible to use the heart rate recovery and the HRV analysis as a simple and noninvasive evaluation method of physiological recovery response following exercise [21,22,23,24]. Presently, there is convincing evidence that some of the protective and therapeutic effects of chronic exercise training are related to its impact on the ANS [25]. Thus, the analysis of the ANS activity in several disorders is particularly helpful for the clinician in order to better characterize the cardiovascular response to exercise and to elucidate the role of physical activity in the relevant disturbance.

Commercial ECG Holter devices allow 24 or 48 h HRV monitoring of subjects with AN; although this represents the most frequently used method in clinical practice, potential technical problems should be considered. First, due to the relatively short monitoring time frame, it may leave some arrhythmias unrecognized [26,27] being its diagnostic yield between 15 and 39% [28]. In addition, this type of ECG monitoring requires wires between the electrodes and the monitoring system itself, which may represent a cause of noncompliance, especially in young subjects, potentially affecting the reliability of the results [29].

In the framework of HR and HRV monitoring, the use of wearable systems combined with wireless technologies can be particularly suitable for monitoring cardiovascular and autonomic parameters during physical exercise, quantifying the autonomic response during such activity, without interferences and possible pitfalls due to the weight of the systems and the use of wires.

We have previously applied wearable technologies for the assessment of the HR and the HRV in young adolescents with AN [30], as well as in other neurodevelopmental disorders [31,32,33]. In a previous study, we recorded the ECG data in resting conditions in subjects with AN and in an age-matched control group while the subjects were lying in a supine position on an ambulatory bed. The acquisition system used was characterized by a chest strap-based wearable solution developed in our Institute in collaboration with SHIMMER^TM^ Research, able to extract a single-lead ECG signal [34]. Thanks to its particular design, with the sensor directly attached to the chest strap through two metal pins, the device guarantees high performances concerning noise and artifact reduction, attributable to the elimination of cables, present in most commercial devices. In our previous investigations, we demonstrated that the wearable sensors combined with the wireless technologies were well accepted in young adolescents with AN [30,35], and we confirmed previous results on autonomic function in these subjects by showing an increased parasympathetic tone at rest [9,14], as compared to control, age-matched subjects.

Among the peculiar clinical characteristics of AN that can be investigated without particular burden for the patients, a high level of physical activity may represent a core feature in a particular subgroup of these patients [36,37]. Indeed, it has been considered not uniquely related to the conscious attempt to lose weight, but deriving from an urge to be physically active and an inability to stay still [38]. This concept—known as “drive for activity” (DFA)—is pivotal in AN as it has been shown that more clinically severe AN conditions are associated to a higher level of DFA [38,39]. However, other investigation identified an enhancing role of physical activity in patients’ weight recovery, especially with regard to body fat [40], or a positive effect of physical activity on nutritional status in acute AN [41].

In a review, El Ghoch and colleagues [42] investigated the link between eating disorders and physical fitness in nonathletes, as well as between eating disorders and sport performance in athletes, displaying a negative effect of eating disorders on both physical fitness and sport performance. Furthermore, several studies reported a general reduction in muscular fitness (i.e., aerobic fitness, musculoskeletal fitness, flexibility, and motor fitness) in patients with a long-term eating disorder [43,44,45,46,47], which is not completely restored after nutritional rehabilitation and weight gain [48]. The main mechanisms involved in this process include low energy availability, excessive loss of fat and lean mass, dehydration, and electrolyte disturbance [49].

However, only few studies have examined the effect of physical exercise on cardiovascular function in patients with AN [29,47,50,51]. These studies showed lower HR, oxygen uptake, blood pressure, workload, and VO_2_, as well as lower left ventricular mass in the AN group with respect to controls, but none of them investigated HRV during physical activity. Given the critical pathophysiological pattern described above, the study of ANS differences between subjects with AN and controls, even during light physical activity, could be of clinical interest to better understand the pathophysiological correlates of specific cardiovascular complications of AN and to identify the AN patients with the highest risk of cardiovascular complications [52].

Therefore, the aims of the present study were twofold: (i) to apply a wearable device previously validated by our group and already tested on a group of young adolescent girls with early onset AN at rest [30] to monitor the HR and the HRV during mild physical activity and (ii) to understand if the response to a mild physical activity is different in adolescents with AN with respect to age- and gender-matched controls.

## 2. Methods

### 2.1. Participants

From June 2012 to February 2014 we recruited and tested 40 adolescent girls. The study population was composed by 23 girls with AN restricting type (AN-r) in line with DSM-IV [53] and DSM-5 [1] criteria, enrolled in the Child and Adolescent Eating Disorders Unit of the IRCCS Stella Maris Foundation (Pisa, IT). Exclusion criteria were the presence of psychotic symptoms, the Wechsler Full Scale IQ **<** 80, the presence of pathologies not related to the eating disorder, current, or previous episodes of substance abuse. Furthermore, we specifically excluded from the study patients with binging and/or purging behaviors. We performed data collection in all subjects within 3 days of patient hospitalization before starting any pharmacologic treatment, if required.

We enrolled a sample of 17 healthy adolescent girls as control group. We recruited the controls among students of middle and high schools in the metropolitan area of Pisa. All control subjects attended regular classes and schools without a support teacher.

The study conformed to the Declaration of Helsinki, and was approved by the IRCCS Stella Maris Institutional Ethical Committee. Each subject and parents or guardian signed an informed consent.

### 2.2. Baseline 12-Lead ECG

We recorded a resting 12-lead ECG in both the AN group and controls at the beginning of each session. We measured QT duration from the beginning of the QRS complex to the end of the T wave, whereas we calculated the corrected QT (QTc) according to the Bazett formula [54].

### 2.3. Echocardiographic Measurements

All subjects underwent a conventional 2D and M-mode Doppler echocardiographic examination, by means of a commercially available instrument (Vivid I, GE Healthcare, Little Chalfont, UK. Echocardiographic images were acquired in the left lateral decubitus position and in the supine position for the subxifoid approach. We used M-mode echocardiography for calculating dimensions of chambers and walls. We measured the left ventricular internal dimensions in end-diastole (LVED) and in end-systole (LVES). Furthermore, we measured interventricular septal wall thickness (IVSWT) and left ventricular posterior wall thickness (LVPWT) in end-diastole according to the American Society of Echocardiography recommendations [55]. We calculated the LV mass index (LVMi) in each subject and related to body surface area. We assessed LV end-diastolic and -systolic volumes and derived ejection fraction by the biplane summation method from the apical four-chamber view [55]. We defined pericardial effusion as the presence of an epicardial to pericardial echo-free space in diastole with a minimum of 2 mm.

### 2.4. ECG Monitoring by Wearable Sensors

We recorded the signals at the IRCCS Stella Maris Foundation (Pisa, Italy) in three different moments. In the first phase (Baseline), we measured signals in supine resting position, with the subject lying on an ambulatory bed. During the 5 min of Baseline, patients and controls were asked to do nothing but relax. Then, they were asked to stand up for 2’ and then to perform the task, consisting of a simple walk of 300 meters along a flat path spaced out by 8 consecutive steps. The duration of the task slightly changed among the subjects from 5 to 6 min. At the end of the task recording we allowed the patient to lie on the ambulatory bed and we acquired the signals for 5 min at rest for the Recovery.

**Figure 1:**
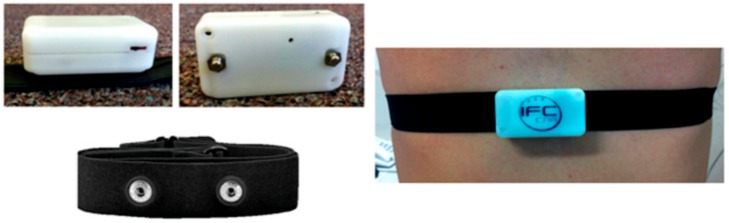
Wearable sensor for electrocardiographic (ECG) signal acquisition based on ECG SHIMMER^TM^ device.

During this protocol, we recorded the ECG signal using a wearable sensing set developed by CNR redesigning the ECG SHIMMER^TM^–based platform, as stated [34] (Figure 1). The device, of extremely small dimension and low weight, includes the electronics for signal conditioning, a power supply based on a 3V Li-ion battery, a 2 GB SD Card for data storage, and data transmission modules (available both via Bluetooth or 802.15.4). The electronic board and its enclosure can be easily plugged in several commercial cardio-fitness chest straps, which are completely waterproof, assuring an ideal and comfortable long-term contact with the thorax, with a self-adaptation to the body shape. The chest belt includes two biocompatible, dry electrodes directly attached to the human skin for single-lead acquisitions without any need for skin preparation, gels, or adhesives employment. The acquisition sampling rate was 200 Hz. We selected this sample frequency to manage the trade-off between battery consumption and accuracy of the signal.

Notably, we previously validated the wearable system used in the present work in a group of healthy subjects [56]. We evaluated the performance of the system at rest and during working activity. In all the scenarios, the chest strap and the gold standard Holter device (ELA medical, Milan, Italy) simultaneously recorded the ECG for 3 h. The resulting waveform confirmed that the signal quality was comparable to that acquired by the ELA Holter. Moreover, the ECG chest strap provided readable signals for more than 95 and 99% of the time of acquisition while the subjects were at work and at rest, respectively. The R peaks detected using the developed algorithm for the ECG chest strap were compared to the ones annotated in a different report provided by the ECG Holter. The mean percentage difference of RR measurements was lower than 10% (maximum value established by the CEI ISO60601-2-47 about RR calculation) during the whole validation. This validation study confirmed that the ECG chest strap system has a high accuracy both at rest and during movement.

In this study, we assessed the possibility of applying the wearable device in subjects with AN during physical activity by the test observation by researchers, as well as by interviews to the subjects at the end of the recovery phase. Each participant verbally answered questions related to the usability of the wearable system, wearability issues and possible discomfort.

### 2.5. ECG Signal Analysis

**Figure 2:**
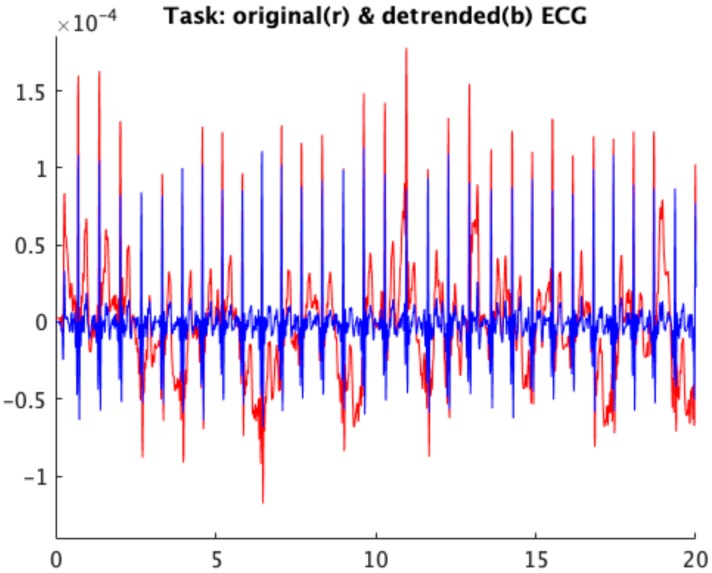
Original ECG signal (red) and signal obtained after preprocessing (blue) for a sample subject during Task.

We processed the ECG signals in MATLAB (The MathWorks, Inc., Natick, MA, USA). More specifically, we analyzed all the 5 min of baseline and recovery, while for the task we selected 5 min of the acquisition for each subject including the steps (the whole registration or the central 5 min in case of 5 to 6 min recordings). First, we applied a stepwise filtering process aimed at removing typical ECG artifacts and interferences. We included a cubic spline 3rd order interpolation between the fiducial isoelectric points of the ECG [57] to remove artifacts due to body movements and respiration. We removed the power line interference and muscular noise using an IIR notch filter at 50 Hz and an IIR low pass filter at 40 Hz. We applied a parabolic interpolation to the signal to avoid the jitter problems induced by the low sampling rate [58,59]. Figure 2 shows the original ECG signal and the signal obtained after preprocessing for a sample subject during the task. Once preprocessed, R waves were detected with the Pan–Tompkins method [60] to obtain the tachogram. Then, we applied an algorithm for the recognition and correction of nonsinusoidal beats as described in [61] to obtain a RR series that only contains variations due to the sinus node and thus reflects the activity of the ANS. We extracted time and frequency domain features from the tachogram according to the International Guidelines of Heart Rate Variability (HRV) [62].

From the tachogram, we obtained the mean HR and several measures of HRV. To this extent, HRV is the result of the influence of the ANS on the heart beat-to-beat fluctuations in the HR, which are mainly determined by the activity of the cardiac sympathetic and parasympathetic systems. In this framework, we performed the HRV analysis using both linear time domain and frequency domain methods.

Within the whole process, we extracted the following time domain measures of heart rate variability: the standard deviation of NN intervals (SDNN), and the root mean square of successive differences (RMSSD). While both parasympathetic and sympathetic activities contribute to the SDNN, the RMSSD is mainly correlated to the parasympathetic branch [63].

For the frequency domain analysis, we calculated the power spectrum density (PSD) using the parametric autoregressive Yule–Walker model [64,65] of order 9 chosen by using the information criterion due to Akaike (AIC) [66]. The features extracted from the PSD and estimated for each frequency band—low frequency (LF: 0.04–0.15 Hz) and high frequency (HF: 0.15–0.40 Hz)—included absolute powers and the LF/HF power ratio. These limits were selected according to the Task Force Recommendations reported in Ref. [62]. We normalized the power of each band to the total power of the spectrum (LFn, HFn). Summarizing, SDNN expresses the overall HRV, therefore the ANS activity; LFn is mainly related to the sympathetic component of the ANS, whereas HFn mainly reflects the activation of the parasympathetic branch of the ANS. The LF/HF ratio indicates the overall balance between sympathetic and parasympathetic systems (see Ref. [63] for more detail).

### 2.6. Statistical Analysis

We performed statistical comparisons of the autonomic function outcome measures using SPSS software (SPSS Inc., Chicago, IL, USA) [67]. We applied the Shapiro–Wilk test to assess the normality of the variables.

We compared demographic with cardiovascular and ANS data at Baseline using Student’s *t*-test when they had a normal distribution and the Kolmogorov–Smirnov nonparametric test when the distribution was not normal. We evaluated the ANS changes during the different phases using a procedure for repeated measurements for comparisons between baseline, task, and recovery phases. In case of normality, we performed a one-way ANOVA procedure for repeated measures. If significant, we applied Student’s post hoc tests for the paired sample. When sphericity assumptions were violated (Mauchly’s test of sphericity), we reported Greenhouse–Geisser corrected *p*-values. We applied Friedman repeated measures ANOVA followed by post hoc analysis with Wilcoxon signed rank test for all other parameters when the corresponding values were not normally distributed. We also compared HR and HRV measures between the two groups in the three different phases. In all these analyses, we used the BMI as a continuous covariate. We accepted statistical significance at *p* < 0.05. We also investigated bivariate correlations between the ECG variables at Baseline and age, BMI or LVMi using Pearson’s or Spearman’s correlation coefficient analysis both in the AN group and in controls. Finally, in the AN group, we studied the correlations between ECG variables at Baseline and the age of onset or the duration of disease.

## 3. Results

### 3.1. Group Characteristics

All subjects completed the cardiovascular examination.

There were no significant between-group differences in age (AN: 15.2 ± 1.9 years, range: 10–19 years; control: 15.7 ± 2.1 years, range: 11–19 years; *p* = 0.4), while, as expected, the BMI was significantly lower in the AN group (AN: 15.7 ± 1.6 kg/m^2^; controls: 21.7 ± 2.8 kg/m^2^; *p* < 0.001). At the same degree, also the associated z-scores related to the BMI taking into account the age of the subjects were significantly different between the AN group and controls (AN: −2.1 ± 1.2; controls: +0.3 ± 0.6; *p* < 0.001). The mean age of disease onset in the AN group was 12.9 ± 2.1 years, while the mean disease duration (from the age of onset to the time of the cardiac evaluation) was 19.1 ± 14.5 months.

The main clinical characteristics of the AN group are summarized in Table 1.

### 3.2. Basal 12-Lead ECG

The mean HR was significantly lower in the AN group than in controls (AN: 58.5 ± 13.2 bpm; controls: 81.5 ± 12.2 bpm; p < 0.001). The QT value was significantly higher in the AN group compared to the controls (AN: 394.5 ± 35.0 ms; controls: 360.5 ± 19.5 ms; p = 0.04), while the QTc was significantly lower in the AN group than in controls (AN: 385.5 ± 32.9 ms; controls 422.5 ± 10.5 ms; *p* = 0.01). Applying the correction for BMI, only the QTc difference remained statistically significant (*p* = 0.04). Notably, the QT and the QTc duration were within the normal range in both groups under study.

### 3.3. 2-D Doppler Echocardiography

All the AN group had a normal left ventricular function (from 60 to 72%, mean: 64%); LVMi was lower in the AN group as compared to controls (AN: 55.67 ± 3.4 g/m^2^; controls: 65.2 ± 8.4 g/m^2^; *p* = 0.01). We detected a mild pericardial effusion, with no evidence of haemodynamic compromise, in nine out of 25 AN subjects. Conversely, none of the control subjects showed signs of pericardial effusion.

### 3.4. HR and HRV Analysis

We completed the ECG acquisition in all the enrolled subjects. No complaints or discomfort were reported by AN patients in terms of usability and wearability of the ECG monitoring tool. The basal comparison between the AN group and controls revealed a significantly lower HR for the AN group (AN: 57.3 ± 16.2 bpm; controls: 81.8 ± 6.4 bpm; *p* < 0.001). At the same time, SDNN was significantly increased in the AN group (AN: 0.16 ± 0.15 s; controls: 0.07 ± 0.07 s; *p* = 0.009), as did RMSSD (AN: 0.11 ± 0.06 s; controls: 0.04 ± 0.02 s; *p* = 0.028).

Frequency domain features were also different between the two groups. Specifically, LFn was significantly lower in the AN group (AN: 0.35 ± 0.14; controls: 0.45 ± 0.14; *p* =0.040), while HFn was significantly higher in these subjects (AN: 0.66 ± 0.15; controls: 0.47 ± 0.17; *p* = 0.004). Consequently, as expected, the LF/HF was significantly lower in the AN group than in controls (AN: 0.83 ± 0.13; controls: 1.16 ± 0.45; *p* = 0.030).

Basal data are summarized in Table 2.

After comparing the two groups at baseline, we evaluated the effect of the task on the autonomic parameters.

HR changed during the different phases of the protocol (F = 18.8, *p* < 0.001), with a significantly increased value of HR among the AN group during the task with respect to the baseline (*p* < 0.001), followed by a similar significant decrease at recovery (*p* < 0.001). No significant changes were otherwise seen among controls.

SDNN was not modified among the AN group (χ^2^(2) = 2.8, *p* = 0.28); on the other hand, it was significantly changed among controls (χ^2^(2) = 12.8, *p* = 0.002), with an increase during the task with respect to baseline (*p* = 0.003), and a following decrease at recovery (*p* = 0.007).

The same trend was seen for RMSSD, with no modifications among the AN group (χ^2^(2) = 2.8, *p* = 0.25), and significant differences in controls (χ^2^(2) = 10.9, *p* = 0.004), with an increase during the Task with respect to the Baseline (*p* = 0.004), and a decrease at Recovery (*p* = 0.009).

The phase-effect was significant for both LFn (F = 9.1, *p* = 0.005) and HFn (F = 5.5, *p* = 0.007) in AN subjects and controls. Among the AN group, LFn significantly increased at task (*p* = 0.005) compared to baseline and equally significantly decreased (*p* = 0.005) at recovery; whereas among controls we reported a significant decrease from baseline to task (*p* = 0.01). We noticed the opposite trend for HFn, which in the AN group significantly decreased at task (*p* = 0.01) compared to the baseline and later significantly increased (*p* = 0.01) during recovery. Among controls, it was significantly increased from baseline to task (*p* = 0.01).

Finally, LF/HF significantly changed among the AN group (χ^2^(2) = 5.1, *p* = 0.010), but not in controls (χ^2^(2) = 1.1, *p* = 0.86). In particular, in AN group, it was increased during the Task, both with respect to the Baseline (*p* = 0.01) and Recovery (*p* = 0.001).

**Figure 3:**
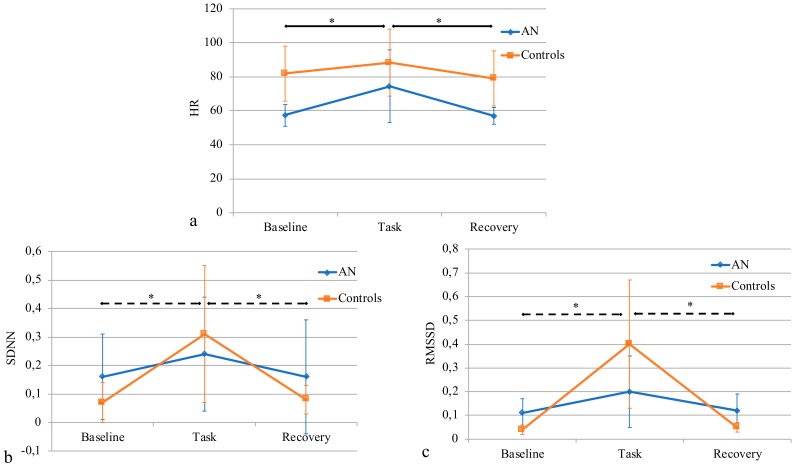
Change in temporal-domain features during baseline, task, and recovery for the AN group and controls. (**a**) Heart rate (HR); (**b**) standard deviation of NN intervals (SDNN); and (**c**) root mean square of successive differences (RMSSD). *: *p* < 0.05; continuous line: significant differences in the AN group; dashed line: significant differences in controls.

**Figure 4:**
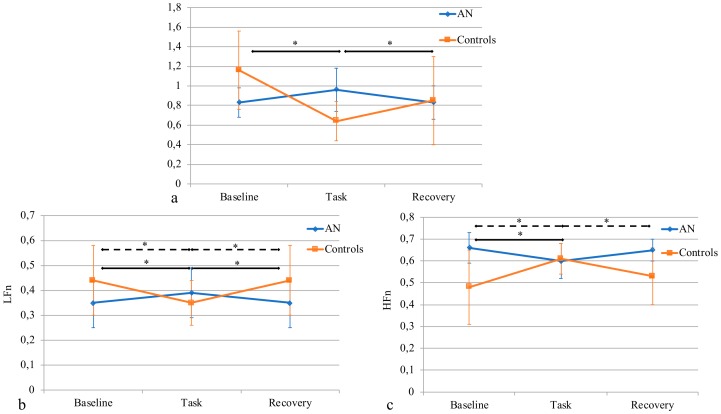
Change in frequency domain features during baseline, task, and recovery for the AN group and controls. (**a**) Low frequency/high frequency ratio (LF/HF), (**b**) normalized low frequency (LFn), and (**c**) normalized high frequency (HFn). *: *p* < 0.05; continuous line: significant differences in the AN group; dashed line: significant differences in controls.

Figure 3 and Figure 4 report the results of a repeated measures ANOVA. 

### 3.5. Correlations

According to the literature, we found a different trend of correlation between BMI and HR in more severe with respect to less severe AN [17]. Thus, we split our sample in two subgroups using the median of BMI (15.9 Kg/m^−2^) as threshold. We noticed a negative, although not significant, correlation between the BMI and the HR in more underweight AN patients (r = −0.44, *p* = 0.18), while we obtained a positive, nearly significant correlation between BMI and HR in less underweight AN subjects (r = 0.66, *p* = 0.05). We failed to find other significant relationships between anthropometric and autonomic measures in both AN subjects and controls.

Concerning cardiac parameters, LVMi showed a significant negative correlation with LFn (r = −0.49, *p* = 0.017) and a significant positive correlation with HFn (r = 0.47, *p* = 0.018) at Baseline in AN group.

## 4. Discussion and Conclusions

The main result of this study points to a significantly different cardiac response during light physical activity between AN subjects and healthy matched controls.

Indeed, at baseline, patients with AN and control subjects had a different autonomic profile, retrieved through the ECG signal, which confirmed our previous results [30] and the findings of other studies performed on adolescents with AN [9,14]. More in depth, subjects with AN displayed a significantly lower HR than controls, consistent with the existing literature, in which bradycardia is widely recognized as one of the prominent physical signs of this psychiatric condition (see, for example [14,17]). We found this trend also during the Recovery, demonstrating that the autonomic effect of physical activity, when existing, is completely reversible in the AN group, which returns to a bradycardic pattern at rest soon after the Task. The bradycardia documented in subjects with AN appears to be related to the severity of this condition, as demonstrated by the correlation between the HR and the BMI, selected as a reliable predictor for AN severity [68]. In the work of Mazurak and colleagues [17], conducted on older females with AN, the authors found a relationship between the BMI and the interbeat interval (IBI). Specifically, patients with lower BMI displayed a positive correlation between the IBI and the BMI, while the opposite trend was detected for BMIs > 17.5. In agreement with this finding, we found an opposite trend between the BMI and the HR in the AN group less underweight with respect to the AN group more underweight. The lack of significance of our results could be partly ascribed to the relatively low sample size of the two subgroups of AN subjects when the whole sample was split according to the BMI. Moreover, bradycardia, reflecting a more profound cardiovascular imbalance of anorectic girls, is associated with more severe stages of AN, thus becoming a good predictor of the clinical stage of the disease [69]. Conversely, age does not appear to influence the ANS functioning, possibly due to the relatively narrow age range included in this research.

It is thought that the physiological adaptation of bradycardia is associated to an increase of the vagal tone and to a decreased energy utilization brought by low calorie intake [70,71]. Those considerations over the vagal tone are somewhat consistent with our results, displaying lower LFn, higher HFn and, consequently, lower LF/HF in the AN group at baseline and recovery. Conversely, we observed opposite patterns among controls.

At baseline, we also noticed an increased QT and a decreased QTc (likely due to significantly reduced HR in AN) in the AN group than in controls. Although the differences were statistically significant both for the QT and the QTc, the values were all within the normal range, thus the clinical significance of these findings is, per se, debatable. Despite the results in literature are controversial, a recent study [72] reported a slightly reduced QTc in subjects with AN restrictive type, in line with our results.

Previous studies have analyzed the influence of the physical exercise on the HR and the HRV measures in physiological and pathological conditions [19,73,74,75,76,77,78], identifying the HR as the easiest parameter to extract for the evaluation of cardiac response to exercise. As is widely accepted, HR is related to oxygen uptake during continuous exercise; in athletes, a reduced increase of the HR during intensive exercise correlates to the degree of fitness of the subjects [79]. In addition, literature findings reported that exercise intensity has a profound influence on the HRV response [19,80,81].

Focusing on the time domain parameters (SDNN and RMSSD), features related to the ANS reactivity, it is worth noting that such parameters are not modified in the AN subjects during the task phase, whereas both of them are increased among controls. The increase in SDNN suggests an overall increase of the ANS activity in healthy subjects during the task, which seems to be driven mostly by an increase in parasympathetic activity. Indeed, the RMSSD, which reflects mostly the activity of the parasympathetic branch of the ANS, also increased during the task. Notably, the level of physical activity demanded in the present protocol was quite light. We performed this methodological choice after consideration of the clinical characteristics of the AN group, already featuring a physiological and nutritional imbalance typical of their condition; in this group, a moderate or intense physical activity was purposefully avoided.

Coherently with the time domain features, our results in the frequency domain could reflect a different response towards a light physical activity in the two groups. In particular, the healthy control subjects showed a slightly increased parasympathetic activity (HF), which may be related to the extremely relaxed conditions of all the girls during the Task, which was considered as a sort of pleasant game to be performed. Other authors have shown the same response in youngsters undergoing a mild physical task in a pleasant environment [82,83].

On the other hand, the AN group showed a slight increase in the sympathetic response (LF) during the task, which represents the sort of physical activity frequently performed in everyday life. Nudel et al. [47] and Bartak and colleagues [84] also obtained similar results, suggesting that in this group of patients even a light task, actually frequently performed during the day, can elicit a cardiac response, as also demonstrated by the increase in the heart rate. This result could be related to the low energy availability due to excessive loss of fat and lean mass.

We observed that wearable sensors and wireless technologies can be applied in the monitoring of adolescents with AN during physical activity without any difficulty experienced by the subjects. Indeed, young adolescent girls with AN did not show sensory–motor and/or behavioral issues in wearing the devices, gave a positive feedback at the end of the test and completed the proposed protocol without problems. In addition, we observed that the signals acquired and preprocessed were stable enough to be analyzed (see Figure 2). Thus, we suggest the possibility to employ the system for the acquisition of ECG signals in motion on subjects who could be threatened by artificial constraints. The possibility of monitoring signals during physical activity could provide more information about the physiological response of the AN subjects with respect to that merely obtained from a clinical observation. This result extends our previous report dealing with ECG recording performed in ambulatory conditions [30], increasing the importance of such approach in a population burdened by risk of lethal arrhythmias (see Ref. [69] for an example).

Although the present investigation may help to improve our understanding of the physiological response to physical activity in young patients with AN-R, the findings must be interpreted in light of some important limitations. First, we did not consider the hyperactivity as a possible symptom associated to the AN psychopathology. Indeed, the sample size was too small to split it into distinct subgroups and, in addition, the definition and the objective quantification of hyperactivity are quite questionable in the literature. Second, we did not use a standard test commonly used in rehabilitative medicine for physical activity (e.g., the 6-Minute Walk Test, 6MWT) since we chose a quite easy natural physical activity, i.e., walking inside the clinic, to let the subjects be as comfortable as possible in performing the protocol. Moreover, although we evaluated the wearable system in a previous study on healthy subjects, we did not validate the device in the present study, i.e., the quality of the signals obtained was not verified. In the future, it could be useful to record the ECG with our system and with a reference device simultaneously, for a comparison of the quality of the signals and of the measure obtained. In addition, our sample was composed of only females; therefore, we could not evaluate gender-specific autonomic response during light physical activity. Later on, the relatively small sample size might represent another limitation of the current study. 

In conclusion, in this study we detected differences in the ANS response between the AN group and the control group. Despite a quite slight effort demanded in this protocol, the different autonomic response we observed could be explained by the diminished exercise capacity already described in the AN subjects and attributed to the excessive weight loss, which in turn leads to reduction of muscle mass and dysfunction of the remaining muscle. A reduction in the cardiac mass, as reported in our AN group, which showed a statistically significant lower left ventricular mass when compared to controls, should also account for the diminished exercise capacity [85,86]. Further investigations in larger groups of AN subjects, with special reference to the restrictive type with hyperactivity traits, are therefore warranted.

## Figures and Tables

**Table 1:** Clinical characteristics of the subjects of the AN group.

Subject	Age (y)	Weight (kg)	Height (cm)	BMI (Kg m^−2^)	z-Score	Age of Onset (y)	Hospitalization (mo)	Duration of Disease (mo)
1	16.5	47.9	177	15.3	−2.83	15.1	16.4	15
2	16.9	39.1	155	16.3	−2.16	15.1	15.6	5
3	13.7	42.8	168	15.2	−1.89	12.3	12.8	5
4	16.7	44.7	162	17.0	−1.66	15.3	16.9	18
5	17.2	42.0	163	15.7	−2.75	12.2	17.2	60
6	18.6	34.3	151	14.9	−2.14	14.7	17.6	35
7	16.2	41.4	170	14.3	−3.65	12.0	14.8	32
8	19.0	37.3	162	17.4	−1.93	16.1	17.1	12
9	14.7	38.9	167	13.9	−3.32	13.1	14.6	17
10	13.4	43.9	160	17.1	−0.56	13.1	13.5	4
11	12.7	42.5	161	16.4	−0.88	12.1	13.2	13
12	17.0	42.6	169	14.9	−3.3	13.2	17.1	47
13	17.0	47.0	166	17.1	−1.7	13.6	17.1	43
14	15.0	34.7	163	13.0	−4.05	14.5	14.11	6
15	13.2	32.0	161	12.3	−4.39	11.8	13.5	21
16	15.8	42.2	151	17.9	−0.95	12.1	12.11	10
17	12.7	39.9	155	16.6	−0.78	11.0	11.11	11
18	13.3	47.3	168	16.8	−0.86	11.6	13.3	21
19	16.0	48.8	166	17.7	−1.12	14.2	15.8	18
20	16.2	41.7	162	15.9	−2.27	14.5	15.6	13
21	10.4	26.4	140	13.5	−2.16	10.0	10.5	5
22	16.2	39.3	160	15.3	−2.65	15.2	16.3	13
23	14.8	54.0	173	18.0	−0.66	13.1	14.1	12

**Table 2:** Baseline comparison between the AN group and controls (*: *p* < 0.05; **: *p* < 0.01).

Feature (mean±SD)	AN	Controls	*p*-Value
HR (bpm)	57.3 ± 16.2	81.8 ± 6.4	<0.001 **
SDNN (s)	0.16 ± 0.15	0.07 ± 0.07	0.009 **
RMSSD (s)	0.11 ± 0.06	0.04 ± 0.02	0.028 *
LFn (n.u.)	0.35 ± 0.14	0.45 ± 0.14	0.030 *
HFn (n.u.)	0.66 ± 0.15	0.47 ± 0.17	0.004 **
LF/HF (ratio)	0.83 ± 0.13	1.16 ± 0.45	0.030 *
